# Partial Resistance of Carrot to *Alternaria dauci* Correlates with *In Vitro* Cultured Carrot Cell Resistance to Fungal Exudates

**DOI:** 10.1371/journal.pone.0101008

**Published:** 2014-07-01

**Authors:** Mickaël Lecomte, Latifa Hamama, Linda Voisine, Julia Gatto, Jean-Jacques Hélesbeux, Denis Séraphin, Luis M. Peña-Rodriguez, Pascal Richomme, Cora Boedo, Claire Yovanopoulos, Melvina Gyomlai, Mathilde Briard, Philippe Simoneau, Pascal Poupard, Romain Berruyer

**Affiliations:** 1 Agrocampus-Ouest, UMR 1345 IRHS, Angers, France; 2 Université d'Angers, UMR 1345 IRHS, SFR QUASAV, Angers, France; 3 INRA, UMR 1345 IRHS, Angers, France; 4 Université d'Angers, UPRES EA921SONAS, SFR 4207 QUASAV, Angers, France; 5 Unidad de Biotecnología, Centro de Investigación Científica de Yucatán, Mérida, Yucatán, Mexico; University of Nebraska-Lincoln, United States of America

## Abstract

Although different mechanisms have been proposed in the recent years, plant pathogen partial resistance is still poorly understood. Components of the chemical warfare, including the production of plant defense compounds and plant resistance to pathogen-produced toxins, are likely to play a role. Toxins are indeed recognized as important determinants of pathogenicity in necrotrophic fungi. Partial resistance based on quantitative resistance loci and linked to a pathogen-produced toxin has never been fully described. We tested this hypothesis using the *Alternaria dauci* – carrot pathosystem. *Alternaria dauci*, causing carrot leaf blight, is a necrotrophic fungus known to produce zinniol, a compound described as a non-host selective toxin. Embryogenic cellular cultures from carrot genotypes varying in resistance against *A. dauci* were confronted with zinniol at different concentrations or to fungal exudates (raw, organic or aqueous extracts). The plant response was analyzed through the measurement of cytoplasmic esterase activity, as a marker of cell viability, and the differentiation of somatic embryos in cellular cultures. A differential response to toxicity was demonstrated between susceptible and partially resistant genotypes, with a good correlation noted between the resistance to the fungus at the whole plant level and resistance at the cellular level to fungal exudates from raw and organic extracts. No toxic reaction of embryogenic cultures was observed after treatment with the aqueous extract or zinniol used at physiological concentration. Moreover, we did not detect zinniol in toxic fungal extracts by UHPLC analysis. These results suggest that strong phytotoxic compounds are present in the organic extract and remain to be characterized. Our results clearly show that carrot tolerance to *A. dauci* toxins is one component of its partial resistance.

## Introduction

Partial or quantitative resistance of plants to pests and diseases has been intensively studied among crops. The prospect of developing a sustainable control method has fostered a tremendous amount of work geared towards identifying the genetic factors determining this resistance (known as Quantitative Resistance Loci, or QRLs) to numerous plant diseases or pests. As a snapshot of this activity, in 2011 alone, 41 papers were published on this topic in *Theoretical and Applied Genetics*, dissecting the determinism of partial resistance to 27 distinct pest species amongst 14 crops. Papers have been published on the subject in that journal every year since 1993, with a peak in 2004 (51 articles). On the other hand, as there is much less data addressing the mechanisms involved in plant pathogen partial resistance, these mechanisms are not clearly understood.

Several reviews on Quantitative Disease Resistance (QDR) have recently been published ([1], [2], [3], [4], [5]). A comprehensive survey of disease resistance mechanisms is presented in some of these reviews. A comparison of major types of plant immune responses (Pathogen Associated Molecular Pattern Triggered Immunity, or PAMP Triggered Immunity or PTI *vs* Effector Triggered Immunity or ETI) suggests that molecular mechanisms of plant-pathogen interactions linked to PTI (basal resistance) and ETI (race specific resistance) share common signaling networks. Similarly, it is quite possible that PTI and ETI share common mechanisms with QDR. With this possibility in mind, Kushalappa and Gunnaiah [6] defined quantitative resistance as the ability of a plant to produce resistance-related metabolites and proteins (also referred to as resistance-related biochemicals) to mitigate the action of pathogenicity factors (enzymes, toxins). The genetic basis of plant resistance is complicated by the existence of different pathogen lifestyles, e.g. necrotrophic, hemibiotrophic and biotrophic agents have been described amongst fungi. Recently, significant progress has been achieved in the understanding of the host response to necrotrophic pathogens, including *Alternaria* species [7]. Plant immunity processes are now better explained through the identification of virulence effectors from fungal necrotrophs and their host cellular targets.

In an excellent review, Poland et al. [5] propose for the first time a classification of the possible mechanisms underlying QDR. Six categories of possible QDR mechanisms underlying observed QRLs were distinguished: (i) QRLs could be linked to genes regulating morphological and developmental traits, (ii) mutations or allelic changes in genes involved in basal defense could have an effect on QDR, e.g. chitin receptor kinase 1 in the *Arabidopsis thaliana*-*Alternaria brassicicola* pathosystem [8], (iii) allelic forms of genes involved in the regulation of signaling pathways, such as the transcription factor WRKY33 in *Arabidopsis* [9], might correspond to QRLs that could modulate resistance levels against necrotrophic or biotrophic pathogens, (iv) QRLs could represent weak forms of major resistance genes (R-genes) or QRLs may colocalize with R-genes (numerous examples, including several plant species in contact with fungal pathogens, are reported in the literature), (v) loci or genes that confer QDR could be components of chemical warfare between the plant host and its pathogen, or (vi) QRLs might represent novel classes of genes, that were not previously described as defense genes supporting resistance mechanisms. Two examples could be mentioned in this latter category: the loss of function of the proline-rich protein Pi 21 is responsible for non-race specific QDR of rice to the hemibiotrophic fungus *Magnaporthe grisea* [10]; and rice indole-3-acetic acid -amido synthetase GH3-2 mediates broad-spectrum partial resistance against two pathogenic bacteria and *M. grisea* by suppressing pathogen-induced auxin production [11].

Since the review of Poland et al. [5], recent advances on determining the mechanisms underlying QDR have been reported in studies involving cultivated monocots of high economic importance. In these studies, specific genes conferring partial resistance to bacterial or fungal pathogens were described: the wheat kinase start protein WKS1 towards the stripe rust pathogen, *Puccinia striiformis* f. sp. *tritici* [12], the wheat serine/threonine protein kinase Stpk-v towards the powdery mildew pathogen *Blumeria graminis* f. sp. *tritici* [13], and the rice putative receptor like cytoplasmic kinase BSR1 towards *Xanthomonas oryzae* pv. *oryzae* and *M. grisea* [14]. In the barley genome, hotspots of non-race specific disease resistance to *Blumeria graminis* were identified with candidate genes encoding components of PAMP-triggered immunity, such as receptor-like protein kinases, factors of vesicle transport and secreted class III peroxidases [15]. In the present paper, QDR will be considered through the involvement of chemical warfare components in the host-pathogen system, as previously suggested by Poland et al. [5]. The production of plant defense compounds in a quantitative or qualitative manner (see for example [16], [17]), or the mechanisms deployed by the plant against pathogen-produced phytotoxins, might contribute to higher partial resistance.

Toxins produced by necrotrophic pathogens, such as *Alternaria* species, have been recognized as important compounds responsible for plant disease, through host cellular death [18]. The capacity of the plant host to resist pathogen-produced toxins via different modes, including detoxification and metabolic bypass, has been extensively described in two pathosystems (*Cochliobolus carbonum*/maize [19]; *Alternaria alternata* f.sp. *lycopercisi*/tomato [20]). In these two examples, toxin resistance mechanisms were described however with respect to qualitative resistance mechanisms. Another example of toxin resistance was reported in the study of Walz et al. [21] using transgenic tomato lines. The introduction of a wheat oxalate oxidase gene in tomato reduced disease symptoms in plants infected by *Botrytis cinerea* or *Sclerotinia sclerotiorum*, two necrotrophic fungi producing oxalic acid, a toxin that is considered to be an important factor determining pathogenicity. In the same line of thought, a correlation between partial resistance and toxin resistance has been found in two other plant-necrotrophic fungal pathogen interactions: *Allium sativum*-*Stemphylium solani* [22] and *Hevea brasiliensis*-*Corynespora cassiicola* (V. Pujade-Renaud, personnal communication). To our knowledge, the discovery of partial resistance mechanisms based on QRLs and linked to a pathogen-produced toxin has never been published. This latter hypothesis is tested in the present paper based on the carrot-*Alternaria dauci* pathosystem.

Phytotoxins produced by necrotrophic fungal pathogens were classified as non-host selective (NHST) and host-selective (HST) toxins. These two toxin categories are respectively related to quantitative and qualitative pathogenicity components [23], but their potential contribution, as aggressiveness factors or factors contributing to the host range, is probably more complex, especially when considering the role of NHST in infection processes. Plant pathogens belonging to the *Alternaria* genus are well-known producers of both types of toxins, most of which are described in different *A. alternata* pathotypes [18]. The necrotrophic pathogen *Alternaria dauci* causes leaf blight, one of the most destructive foliar diseases in cultivated carrot. Brown lesions formed on leaves are often surrounded by a chlorotic halo probably due to the action of one or several toxins. This fungus may produce NHST and HST, but literature concerning the toxin produced by this species is relatively scarce. Papers concerning this pathosystem are mainly focused on the characterization of zinniol, which is assimilated as an NHST. Zinniol could exert its phytotoxic activity through disturbance of membrane due to its effect on calcium channels [24], [25]. It was previously demonstrated that different plant pathogen species of *Alternaria* (generally species exhibiting large conidia with a long beak) and the sunflower pathogen *Phoma macdonaldii* can produce zinniol [26], [27]. In a recent study dealing with the *Alternaria tagetica*-marigold (*Tagetes erecta*) pathosystem, the classification of zinniol as a phytotoxin was however controversial [28]. By comparison to other NHSTs, high zinniol concentrations are indeed required to obtain phytotoxicity in *T. erecta* cell cultures.

Other secondary metabolites synthesized by *A. dauci* have been described, such as alternariol or alternariol monomethyl ether [29], [30], which are also known as mycotoxins synthesized by *Alternaria* species in tomato [31]. It was suggested that alternariol produced by *A. alternata* acts as a tomato tissue colonization factor [32]. Among secondary metabolites of *A. dauci*, four unknown species-specific compounds were reported [29]. The phytotoxin role of these unknown compounds was not specified and remains to be clarified. In a previous paper, we showed that, in greenhouse conditions, the studied host range of *A. dauci* was not restricted to cultivated carrot [33]. Lesions varying in severity and extent were indeed observed on wild *Daucus* species, different cultivated Apiaceae species, and also on all tested dicotyledonous species, such as tomato or radish. Thus, *A. dauci* can exhibit a broad host range in controlled conditions, which suggests that HST production does not have an important role in the biology of this species.

The aims of the present work were to (i) determine if the partial resistance of carrot to *A. dauci* could at least partly be based on resistance mechanisms against toxic metabolites produced by the fungus and (ii) better characterize those metabolites. Carrot has been used as a model plant for somatic embryogenesis studies since the discovery of this regeneration pathway [34]. Carrot is thus very well adapted for *in vitro* studies using plant cells and tissues [35], [36]. Embryogenic cellular cultures were obtained from carrot genotypes with varying degrees of resistance to *A. dauci* and were confronted with fungal exudates. Two levels of response were analyzed: (i) cytoplasmic esterase activity which was previously used as a marker of cell growth and viability [37] and (ii) the differentiation of embryogenic cells to somatic embryos (globular, heart-shaped and torpedo-shaped embryos) in auxin-depleted culture medium. We also confronted these cultures with synthetic zinniol at different concentrations, aqueous and organic fungal extracts. Moreover, zinniol concentrations in fungal extracts, and its chemical stability in our experimental conditions were evaluated. Our results suggest that carrot tolerance to *A. dauci* toxic metabolites is one important component of the partial resistance in this pathosystem. It was also demonstrated that the phytotoxic activity is not caused by zinniol, but instead is linked to the organic phase obtained from the fungal exudates.

## Materials and Methods

### Plant and fungal material, inoculation and symptom scoring

The *Daucus carota* genotypes used in this study were Bolero, Presto, K3, I2, H4 and H1. Bolero and Presto are Nantaise type hybrid cultivars used as standards for resistance and susceptibility, respectively, as in [17], [38], while K3, I2, H4 and H1 are breeding material. H1 plants were obtained by self-pollinating a single plant of a susceptible S3 line obtained from French genetic background at Vilmorin (France). I2 and K3 were obtained in the same fashion from two partially resistant Asiatic lines both developed at Agrocampus Ouest (Angers, France). I2 and K3 are genetically different according to preliminary molecular studies (Le Clerc et al., submitted). H4 was obtained from a partially resistant South American cultivar. All fungal material used in this study was from the *A. dauci* reference strain FRA017, which was also used in previous studies [33], [38], [39]. This strain was isolated in 2000 from naturally infected carrot leaves collected in Gironde, France.

All plant cultivation and inoculation procedures have already been described in detail in [39] (plant cultivation) and [38] (fungus cultivation, inoculum production, drop inoculation). Briefly, plants were grown in greenhouse conditions in boxes containing peat moss/sand mixture for 6 weeks. *Alternaria dauci* was grown in petri dishes on V8 agar, incubated at 24°C in darkness for 7 days, and then exposed to near-ultraviolet light for 12 h/day for 10–15 days for conidia production. The conidia suspension concentration was adjusted to 200 conidia mL^−1^ in 0.05% Tween 20. Individual L3 leaves were inserted in an incubation chamber without being detached from the plant, and forty 5 µL drops of inoculum were applied using a micropipette. The symptom number was evaluated at 7, 9 and 13 dpi and is expressed as the number of symptoms per conidia. The areas under the disease progression curve (AUDPC) were calculated from these data. Leaves were then harvested for qPCR analysis. qPCR evaluation of *A. dauci* in carrot leaves has already been described [38]. Briefly, fungus genome copy numbers (N_f_), evaluated by qPCR from 25 ng DNA samples, were used to calculate infection ratios I = 100×N_f_/N_p_, as described in Berruyer et al. [40], where N_p_ stands for carrot genome copy number. For each genotype, the experiment was repeated four to five times, with each repetition consisting of four inoculated leaves.

### Fungal extract preparation

Fungal extracts were prepared from liquid cultures. Erlenmeyer flasks (250 mL) containing 100 mL of liquid carrot juice medium [Joker 100% pure carrot juice (Eckes-Granini Group GmbH, Nieder-Olm, Germany): 20% v/v, CaCO_3_: 3 g L^−1^; pH 6.8; H_2_O: q.s.p. 1 L] were inoculated with a conidial suspension to reach a final concentration of 5.10^3^ conidia mL^−1^. The fungus culture was grown in the dark for 48 h at 24°C on an orbital shaker set at 125 rpm. Liquid phase (raw *Alternaria* extract, rA) was recovered by filtration through Sefar Nitex (Sephar AG, Heiden, Switzerland) nylon membranes of the following decreasing porosities: 200 µm, 11 µm and 1 µm. Organic compounds were derived from the raw extract by liquid-liquid extraction. pH was adjusted to 7 and one volume of ethyl acetate was added to one volume of raw extract. The mixture was strongly agitated, left to rest, and the phases were separated in a separating funnel. The operation was repeated thrice; the organic phases were pooled and labeled organic *Alternaria* extract (oA). The remaining aqueous phase (aqueous *Alternaria* extract, aA) and the raw extract were freeze dried, weighed and stored in a dessiccator. The organic phase was dried over sodium sulfate, filtered and evaporated under reduced pressure using a rotary evaporator (Rotavapor Büchi Labortechnik AG, Flawil, Switzerland) with a water bath at 25°C, weighed and stored at −20°C. Typical yields were of 13 mg mL^−1^ for the raw extract and aqueous phase, and 30 µg mL^−1^ for the organic phase. Mock extracts (raw, organic and aqueous, respectively labeled rM, oM and aM) were obtained with similar yields from mock cultures incubated in the same conditions. Fungal extracts were also prepared from cultures grown in liquid V8 medium for four days in similar conditions, or in anoxic conditions (12 days at 24°C without shaking).

### Zinniol synthesis and conservation

We wanted to develop a safer zinniol synthesis procedure by reducing the use of toxic reagents such as zinc cyanide and hydrogen chloride gas during the formylation step. Unfortunately, we were unable to modify the previously reported strategy under any of the tested experimental conditions. Therefore the synthetic zinniol samples used in this study were prepared using the approach developed by Martin and Vogel [41]. All the spectroscopic data were in accordance with those reported in that paper. Proton Nuclear Magnetic Resonance (^1^H NMR) analyses were performed in deuterated solvents or a mixture of solvents (Deuterated chloroform, or CDCl_3_; Dimethyl sulfoxide, or DMSO; deuterium oxide, or D_2_O) using a JEOL GSX270WB spectrometer. Stability of zinniol was studied in CDCl_3_, deuterated DMSO-aqueous buffer at pH 5.6, and B5 Gamborg medium [42]. For stability in B5 Gamborg medium, the solutions were sampled at different times, and the samples were stored at −80°C. High Pressure Liquid Chromatography (HPLC) analyses were performed on a Waters 2695 separation module coupled to a Waters 2996 Photodiode Array (PDA) Detector using the Empower software package. A QK Uptisphere 3ODB RP18 column (150×4.6 mm, 3 µm, Interchrom) was used for organic extract analysis with the following gradient: initial mobile phase MeOH/H_2_O 10/90 reaching 100/0 (v/v) in 25 min, with a 0.7 mL min^−1^ flow rate.

### Zinniol detection

Tandem ultra high-performance liquid chromatography- mass spectroscopy (UHPLC-ESI-MS) analyses allowed us to determine the detection level and the amounts of zinniol in different *A. dauci* cultures extracts. Dried extracts of *A. dauci* cultures were extemporaneously dissolved in ethyl acetate/methanol (50∶50, v/v) at a working concentration of 6.67 mg mL^−1^ and filtered through a 0.2 µm nylon membrane prior to immediate analysis by UHPLC. These analyses were performed using an Accela High Speed LC System (ThermoFisher Scientific) consisting of a quaternary pump with an online degasser, autosampler, PDA detector and a TSQ Quantum Access MAX triple stage quadrupole mass spectrometer with an ESI interface. The chromatographic analysis was achieved on a Agilent Zorbax Eclipse Plus C_18_ reversed-phase analytical column (2.1×100 mm×1.8 µm). An elution gradient of water (Milli-Q quality) and acetonitrile (LC–MS grade) was used. Two microlitres of each *A. dauci* culture extract or standard zinniol solution were injected using the partial loop injection mode (10 µL loop size). The PDA detector was set in the 200–500 nm wavelength range with two selected channels at 210 and 233 nm. Data were acquired and processed using the Xcalibur 2.0 software package (ThermoFisher Scientific). Standard zinniol solutions were freshly prepared to obtain five concentrations in the 0.05–5 mg mL^−1^ range.

### In vitro culture methods

Plants were grown in greenhouse conditions for 2 months as previously described. For callogenesis induction, petiole explants (10 cm) were surface disinfected for 5 min with ethanol at 70% (v/v), followed by immersion in a 25% (v/v) commercial bleach solution for 20 min and subsequently washed three times with sterilized twice distilled water. Petioles were sectioned (1 cm) and placed in Petri dishes containing solidified B5 Gamborg medium [42] supplemented with 30 g L^−1^ sucrose, and 0.5 mg L^−1^ 2,4-dichlorophenoxyacetic acid (2,4-D) and 7 g L^−1^ agar. The pH was adjusted to 5.8. The cultures were maintained at 23°C (16 h) and 19°C (8 h) in the dark. In order to induce embryogenic callus development, calli were separated from the original petiole material and propagated by subculturing every 6 weeks in solidified B5 Gamborg medium (macronutrients diluted for ¾) supplemented with 0.1 mg L^−1^ 2,4-D.

For the embryogenic suspension cell cultures, 1 g of friable calli was transferred to a Corning flask containing 25 mL of B5 Gamborg liquid medium (hereafter called “B5 medium”). The medium was supplemented with 0.25 mg L^−1^ 2,4-D and 0.05 mg L^−1^ kinetin to maintain cells in a dedifferentiated state. The cultures were maintained under continuous agitation (125 rpm) at 22°C in the dark. After 3 weeks, cells were separated from calli by sieving through 450 µm mesh pore sieves (Laboratory sieves Ø45 mm; Saulas, France). Cells were retained on nylon membrane (50 µm pore diameter: Sephar Nitex) and transferred on the same fresh medium for 2 weeks of culture. For somatic embryo development, cells were sieved through 200 µm mesh pore sieves. Cells retained on nylon membrane (25 µm pore diameter) were transferred onto 12.5 mL of the B5 medium without growth regulators. In the absence of growth regulation factor, carrot cells spontaneously undergo embryogenesis.

### Embryogenic cell treatments

Lyophilized raw and aqueous fractions were resuspended in growth regulator-free B5 liquid medium in the same proportion (w/v) prior to lyophilization. Organic fractions were resuspended in DMSO and then diluted in growth regulator-free B5 liquid medium. For all fractions, after the pH was adjusted to 5.8, solutions were filter sterilized and kept at −20°C until use. Zinniol (2 mM) was prepared in DMSO (0.4%) and growth regulator-free B5 liquid medium. Cells in the 25–200 µm size range were recovered by filtration and allowed to recover overnight at 22°C in the dark, under shaking at 125 rpm, in growth regulator-free B5 liquid medium. One mL of cell suspension was distributed into each well of enzyme-linked immunosorbent assay (ELISA) plates, and then one mL of fungal extract solution in growth regulator-free B5 liquid medium was added in order to reach final concentrations of 25% (v/v) of the original culture medium in which the fungus had been grown. After adding the extracts, cell incubation was continued under continuous agitation (125 rpm) at 22°C in the dark. When needed, cells were transferred weekly into fresh growth regulator-free B5 medium containing the same extracts. Cell treatments with zinniol at 0.025 µM (z1), 10 µM (z2) and 500 µM (z3) were performed the same way. DMSO 0.4% in growth regulator-free B5 liquid medium was used as mock extracts. The whole experiment was repeated at least three times per condition.

### Fluorimetric evaluation of cell esterase activity

Enzymatic assays were conducted following protein extraction performed according to Vitecek et al. [43] with some modifications. For each condition, 1 mL of cultured cells was collected and centrifuged at 1 800 g for 10 min at 22°C. The supernatant was removed and replaced with 500 µL of 50 mM potassium phosphate buffer (pH 8.75). After centrifugation at 7 200 g for 10 min at 22°C, the pellet was resuspended in a 2 mL microtube in 100 µL of 250 mM potassium phosphate buffer (pH 8.75) containing 1 mM dithiothreitol. Then a thin spatula tip of Fontainebleau sand and one 4-mm diameter stainless steel ball were added. Each sample was frozen in liquid nitrogen, and then ground twice in a Retsch MM301 laboratory ball mill for 30 s at 30 Hz. After grinding, 100 µL of 250 mM potassium phosphate buffer was added to each sample. The homogenate was then centrifuged at 4°C for 15 min at 10 000 g. The supernatant (200 µL) was collected, frozen in liquid nitrogen and stored at −80°C until further use.

The enzymatic assays were performed at a final volume of 300 µL in 96 well ELISA plates. In each well, 20 µL of supernatant was added to 200 µL of 1 M potassium phosphate buffer at pH 8.75. The reaction was started by adding 80 µL of buffer supplemented with fluorescein diacetate (FDA) at 5 µM final concentration from a 1 mg mL^−1^ stock solution of FDA in acetone stored at −80°C. Twenty µL of extraction buffer was used as a blank. The enzymatic reaction kinetics were recorded using a FLUOstar Omega (BMG Labtech) plate spectrofluorometer set to detect fluorescein fluorescence (excitation wavelength: 485 nm, emission wavelength: 520 nm) for 90 min at 45°C. The fluorescein concentration was calculated by comparing the fluorescence data with a standard curve as in Green et al. [44]. Enzyme activity was expressed in nmol fluoresceine min^−1^ and specific activity in nmol fluorescein min^−1^ mg protein^−1^. The protein concentration in samples was measured by the method of Bradford [45] with a commercial protein assay kit (Sigma-Aldrich). In the case of cultivar Presto, protein concentrations were too low to accurately calculate specific activity.

### Microscopic evaluation of cell viability and embryogenesis ability

The ability of cells to differentiate and develop somatic embryos was monitored for 3 weeks after treatments. Proembryogenic masses and somatic embryo formation were visually checked under a stereo microscope (Olympus SZ61TR) fitted with a digital camera (Olympus DP20). Membrane integrity and cell viability were evaluated by a modified double staining method [43] using fluorescein diacetate (FDA) and propidium iodide (IP). In living cells, FDA is degraded into fluorescein, a green fluorescent compound that cannot escape the cell. IP can only enter dead or dying cells through damaged plasma membranes. An FDA stock solution (1 mg mL^−1^ in acetone) was maintained at −80°C, and was extemporarily diluted 10-fold in bi-distilled water (working solution). IP 0.15% was prepared in a phosphate buffered saline solution and maintained at 4°C in the dark. One drop of the cell suspension was placed on a microscope slide and 15 µL of IP and FDA working solutions were added. After 5 min incubation in the dark at Room Temperature (RT), stained cells were observed under a fluorescence microscope (Leica DMR HC) fitted with a digital camera (Qimaging, Retiga 2000R) and monitored using Image Pro Express 6.0 software. Green and red fluorescence indicated viable and dead cells, respectively.

### Statistical analysis

All statistical analyses were performed using R-2.6.1 software (R Development Core Team, 2005). Symptom scoring and qPCR data were analyzed as in [38]. Briefly, log(AUDPC) and log(I+1) were subjected to analysis of variance (ANOVA) and Waller-Duncan multiple comparison procedures. Specific activity data were analyzed as follows: first, the whole dataset was subjected to ANOVA followed by multiple comparisons. The specific activities revealed homoscedasticity (residual vs fitted plot), but a cultivar effect on the residual distribution was observed (residuals vs cultivar box plot). Thus, a separate ANOVA followed by multiple comparisons were also performed for each cultivar. Regardless of the method used, in some instances, mock extracts and DMSO revealed significant effects compared to control. In order to isolate the *A. dauci* exudate and toxin effects from the fungal growth medium and solvent effects, specific activity ratios (rA/rM, oA/oM, aA/aM, z1/DMSO, z2/DMSO and z3/DMSO) were calculated for each independent experiment. For each cultivar × treatment combination, 6–12 figures were calculated from independent repetitions. These results were analyzed by ANOVA using the cultivar × treatment combination as a factor. A 95% confidence interval was calculated in order to check for significant activity variations. When 1 was not included in the interval, the variation was considered significant. Correlations between relative activities were calculated by comparing mean ratios for each cultivar.

## Results

### Evaluation of plant resistance to fungal disease

**Figure 1:**
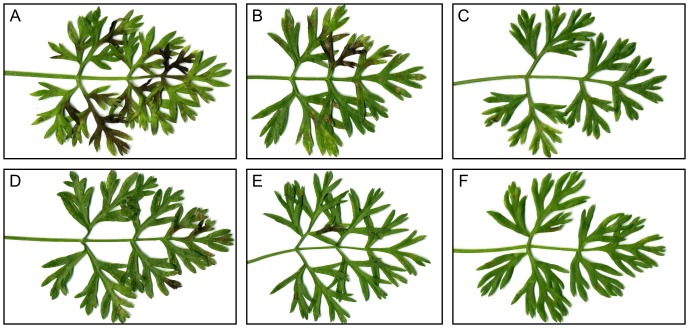
Range of symptoms observed on leaves 13 days after inoculation. The symptom number was assessed at 7, 9 and 13(see Table 1). The leaves shown here show a symptom severity representative of the plant partial resistance level. **A**: H1, **B**: Presto, **C**: K3, **D**: H4, **E**: Bolero, **F**: I2. H1, K3, H4 and I2 are breeding lines, while Presto and Bolero are widely cultivated Nantaise type carrot cultivars.

Six carrot genotypes representative of a broad spectrum of levels of resistance to *Alternaria dauci* were used in this experiment. They included Presto and Bolero, standard cultivars used respectively for susceptibility and resistance towards *A. dauci*. In previous greenhouse and field resistance tests (Le Clerc et al., submitted), Presto and H1 were found to be susceptible to *A. dauci*, while Bolero, I2 and K3 were found to be more resistant. H4 showed intermediate resistance levels. These genotypes were challenged with *A. dauci* using the drop inoculation method as in [38]. The log(AUDPC) was calculated via visual scoring, and log(I+1) by qPCR evaluation of the fungal biomass. As could be expected, there was a close correlation between the two parameters (r^2^ = 0.793, see Table 1). Interestingly, the log(AUDPC) seemed to show a higher resolution, as the homogeneity groups appeared to be more numerous (4 vs 2). The resistance classification obtained in this experiment was similar to the findings of previous field and greenhouse experiments. H1 was found to be significantly more susceptible than Presto. H4 was found to be significantly more resistant than Presto, but significantly more susceptible than Bolero. K3 and I2 did not show any significant difference in resistance level with Bolero (Table 1, Fig. 1).

**Table 1:** Comparison of two different carrot *A. dauci* colonization evaluation methods, symptom number assessment and qPCR-based fungal biomass evaluation.

	log (AUDPC)		log(I+1)	
genotype	mean	homogeneity groups^1^	mean	homogeneity groups^1^
H1	3.09	a	0.79	ab
Presto	2.92	b	0.91	a
H4	2.71	c	0.48	c
I2	2.56	cd	0.51	bc
Bolero	2.53	d	0.39	c
K3	2.46	d	0.36	c

Carrot plants of six different genotypes were tested for *Alternaria dauci* resistance using two different methods simultaneously. Plants were grown in greenhouse conditions. The third leaf was inoculated after it was isolated in an incubation chamber without detaching it from the plant. The symptom number was assessed at 7, 9 and 13 dpi. At 13 dpi, leaves were detached and then subjected to DNA extraction and qPCR for fungal biomass evaluation. Log(AUDPC) was calculated from the visual assessments, log(I+1) from the qPCR experiments. Both were subjected to variance analysis followed by a Waller-Duncan multiple comparison. As could be expected, the two parameters were closely correlated (r^2^ = 0.793). Interestingly, log(AUDPC) seemed to show a higher resolution, as the homogeneity groups appeared to be more numerous (4 vs 2).

1 Homogeneity goups were calculated using the Waller-Duncan multiple comparison following an ANOVA analysis.

### Zinniol synthesis, stability and concentration in fungal extracts

**Figure 2:**
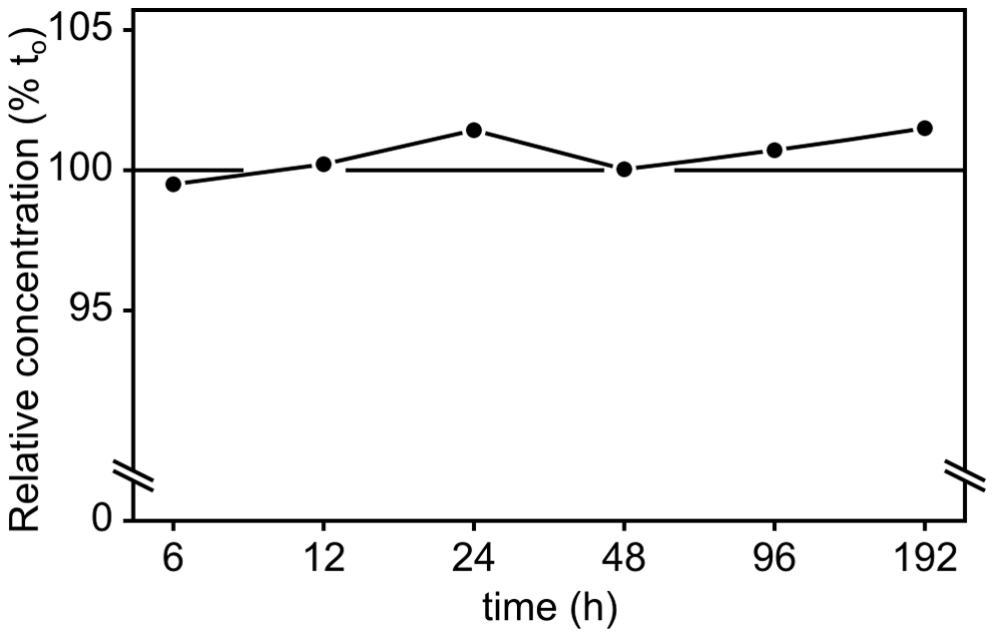
Stability of zinniol over time. Synthetic zinniol was added to Gamborg medium in order to check its stability over time under our experimental conditions (dark, 22°C, shaking). HPLC was used to measure variations in the zinniol concentration over a time course. Three different HPLC analyses were performed for each time. Zinniol concentrations were divided by the initial zinniol concentration in the medium, giving a relative zinniol concentration (noted % t_o_). Except for small (less than 2%) random variations, the zinniol concentration did not vary over time, indicating stability. Standard errors are not represented because they were smaller than the dots we used.

In our hands, the NMR samples of zinniol in CDCl_3_ proved to be rapidly degraded at room temperature after a few days (Fig. S1). This major stability issue encountered during its analysis raised questions on its storage and extraction from fungal culture filtrates. Many papers have reported the use of chloroform as solvent to both extract and store zinniol produced by *Alternaria* fungi [41], [46]. We suspected that the potential residual acidity of this solvent was the main factor explaining this pattern. As the culture medium used for *in vitro* cultures (B5 Gamborg medium) is about pH 5.8, we aimed to determine the stability of zinniol in these conditions. HPLC analysis proved that zinniol was stable at pH 5.6 in a deuterated DMSO-aqueous buffer solution after 1 week at RT (Fig. S1). We then used HPLC to determine the stability of zinniol in the *in vitro* culture medium over 7 days (Fig. S1 and Fig 2). As no significant zinniol variations were observed between samples, we concluded that the compound was stable in the culture medium conditions used in this study.

**Figure 3:**
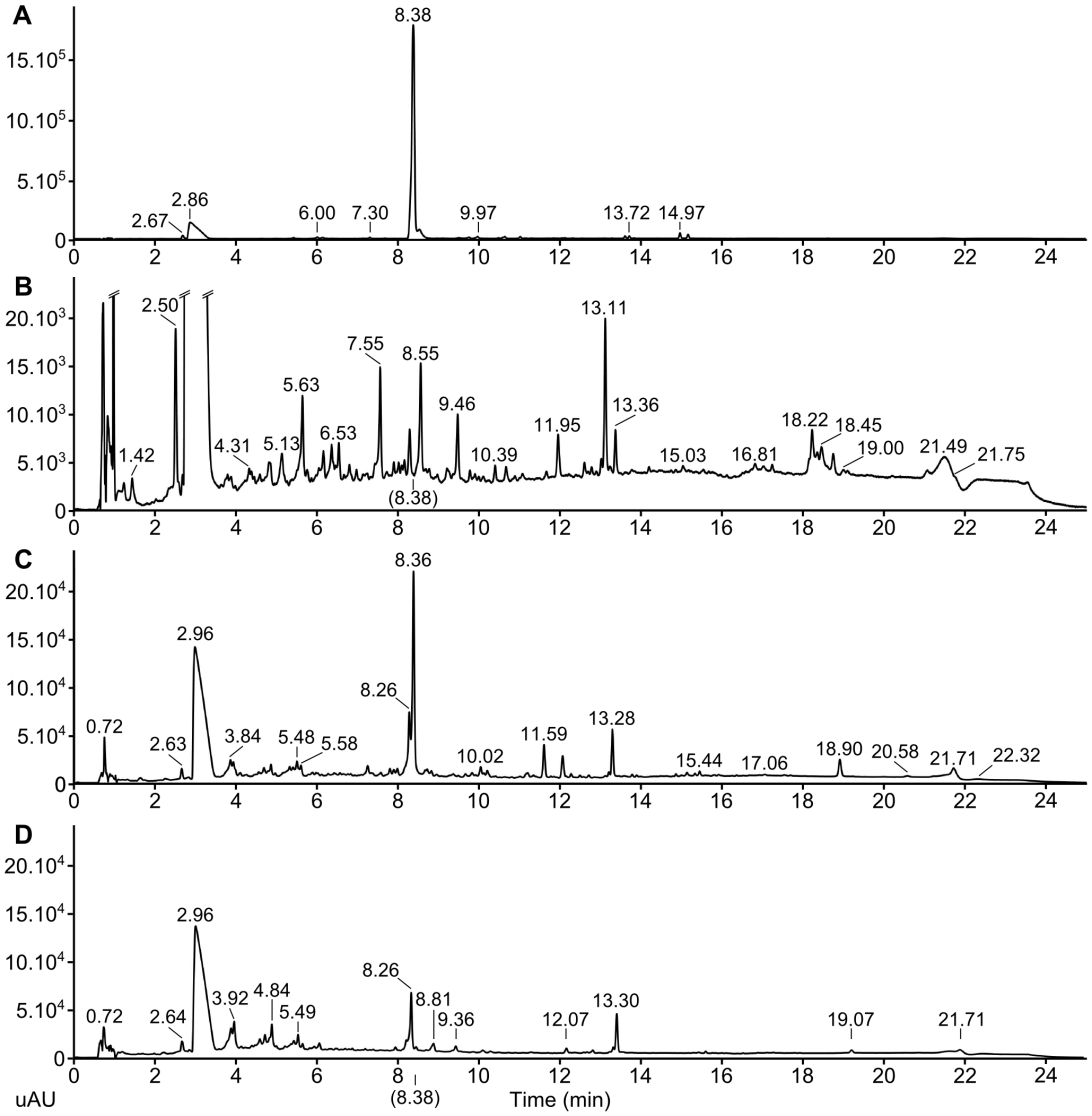
UHPLC detection of zinniol in fungal extracts. UHPLC chromatograms were obtained from different FRA017 *Alternaria dauci* fungal extracts and compared with an UHPLC chromatogram of pure synthetic zinniol. Retention times corresponding to main peaks are indicated **A**: UHPLC chromatogram of 10 µg synthetic zinniol. Observed zinniol retention time is 8.38 minutes **B**: UHPLC chromatogram of 13.4 µg organic extract of an *A. dauci* culture after 48 h under shaking conditions in carrot juice medium. Zinniol expected retention time of 8.38 minutes is indicated. **C**: UHPLC chromatogram of 13.4 µg organic extract of an *A. dauci* culture after 12 days without shaking (anoxic conditions) in V8 medium. A strong peak is visible, corresponding to zinniol retention time. **D**: UHPLC chromatogram of 13.4 µg organic extract of an *A. dauci* culture after 48 h under shaking conditions in V8 medium. Zinniol expected retention time of 8.38 minutes is indicated. Chromatograms C and D have the same scale. uAU: micro Absorption Units (optical density) at 233 nm.

Zinniol concentrations in fungal organic extracts were evaluated by UHPLC-MS. No significant amounts of zinniol were found (Fig. 3B compared to Fig. 3A). Based on the injected quantity of the fungal organic fractions, we concluded that the zinniol concentration was below 0.075% w/w in these fractions, which corresponded to 100 nM zinniol in the fungal growth medium. In order to check if the absence of zinniol was due to the genetic background of fungal strain FRA017 or to the culture conditions, FRA017 was grown in V8 liquid medium in the same conditions, and once again, no significant amounts of zinniol were found (Fig. 3D). Furthermore, the fungus was grown in V8 liquid medium for 12 days in anoxic conditions. In the corresponding organic extract, a zinniol concentration of about 4% w/w (corresponding to roughly 5 µM) was detected (Fig. 3C). The detection of significant amount of zinniol in the organic extract is thus dependent on the fungal culture condition: anoxic conditions seem to be needed.

### Plant cell resistance to Alternaria exudates and zinniol: cell somatic embryogenic ability

**Figure 4:**
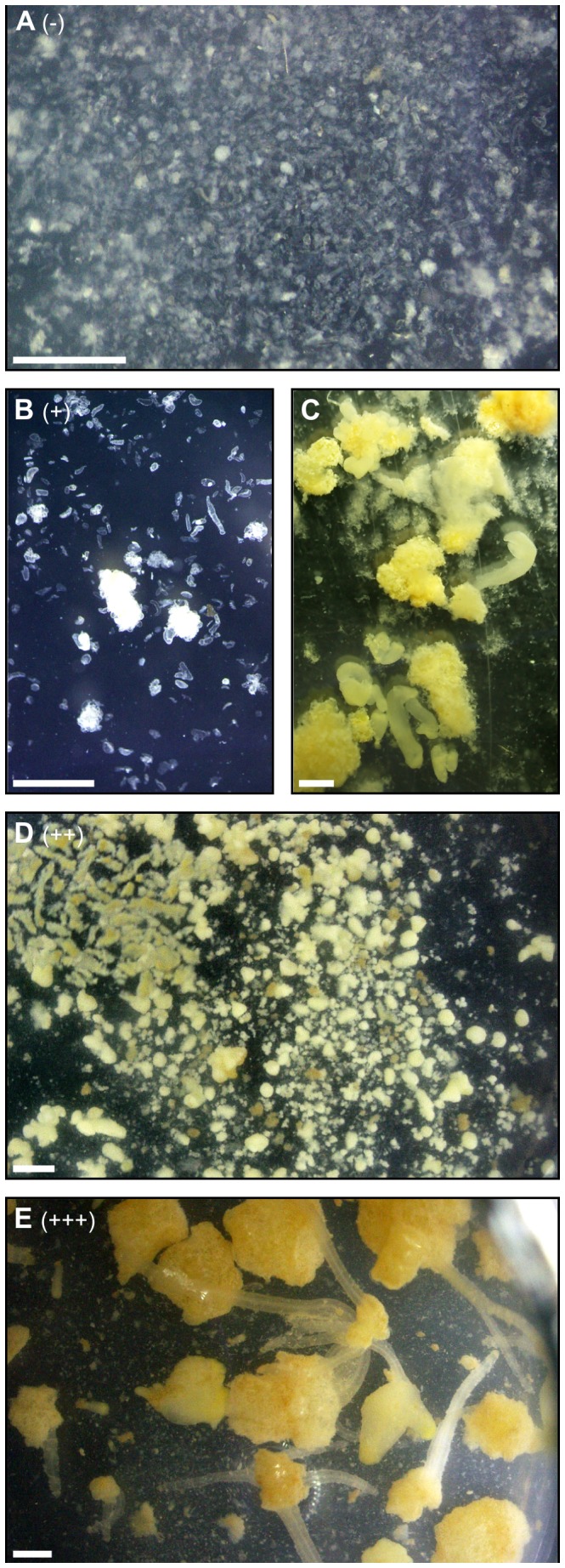
Range of embryogenic activity observed in cell suspensions 3 weeks after treatment. In order to assess carrot cell resistance to fungal toxins, carrot cell suspensions were tested for embryogenesis in the presence of fungal extracts and toxins. Embryogenesis was assessed 3 weeks after treatment, and compared to negative controls. Four levels of embryogenic activity were noted. **A**: (−) no embryogenesis was visible, cells were damaged, **B**: (+) early-stage embryogenic masses were visible, **C**: same as B, but after 6 weeks. **D**: (++) embryos were present, and **E**: (+++) embryogenesis was profuse.

Bolero, Presto, I2, K3, H1 and H4 cultured cells were challenged with various fungal, zinniol, and carrot juice medium extracts. Treatments with DMSO (0.1%), fungal growth medium raw (rM), aqueous (aM) and organic (oM) extracts yielded similar results: as untreated cultures (control), and regardless of the genetic background, cells survived well after treatment and underwent embryogenesis 3 weeks later (Fig. 4, Table 2).

**Table 2:** Influence of cultivar, fungal exudate fractions and zinniol on cell suspension integrity and somatic embryogenesis.

Treatment	Carrot genotype					
	Bolero	H1	H4	I2	K3	Presto
rA^1^	++^2^	−	−	+++	++	−
rM	++	+	+	+++	++	+
aA	++	++	+	+++	++	+
aM	++	++	+	+++	++	+
oA	+	−	−	++	++	−
oM	++	+	+	++	++	+
C	++	++	+	+++	++	+
DMSO	++	+	+	+++	++	+
z1	++	+	+	+++	++	+
z2	++	+	+/−	+++	++	+
z3	−	−	−	+	+/−	−

Carrot cell suspensions with six different genotypes were tested for embryogenesis in the presence of fungal extracts and toxins. Embryogenesis was assessed 3 weeks after treatment.

1 Treatments were as follows: rA: *Alternaria dauci* (strain FRA017) fungal culture raw extract; rM: uninoculated medium raw extract; aA: *A. dauci* fungal culture aqueous extract; aM: uninoculated medium aqueous extract; oA: *A. dauci* fungal culture organic extract; oM: uninoculated medium organic extract; DMSO: DMSO solution, at a concentration corresponding to oM, z1, z2 and z3 treatments; z1: 0.025 µM zinniol; z2: 10 µM zinniol; z3: 500 µM zinniol. C: no treatment.

2 The signs are as follows: (−) no embryogenesis was visible and cells were damaged, (+) early-stage embryogenic masses were visible, (++) embryos were present, (+++) embryogenesis was profuse. +/− early-stage embryogenic masses were visible, or no embryogenesis was visible depending on the repetition.

Three weeks after adding the fungal extract (rA), H1, Presto and H4 cells showed marked damage, with the presence of a high quantity of cell debris, while Bolero, I2 and K3 cells formed embryos in a fashion that could not be distinguished from the controls (Fig. 4, Table 2). Similar results were also obtained 3 weeks after treatment with the fungal organic fraction (oA): Bolero, I2 and K3 cell suspensions underwent embryogenesis, H1, Presto and H4 cell suspensions did not undergo embryogenesis, and showed substantial amounts of cell debris. Conversely, no effects were observed when cell suspensions were treated with fungal aqueous fractions (aA): 3 weeks after treatment, no difference was noted between the treatments and controls (Fig. 4, Table 2). Concerning zinniol, no cultivar differential effect was observed. When 0.025 µM or 10 µM zinniol was added (treatments z1 and z2), no difference was noted between the treated cells and controls, irrespective of the genetic background. At 500 µM zinniol (z3), cell suspensions formed debris, and no embryogenesis was observed 3 weeks after treatment. Both susceptible and resistant cultivars were affected (Fig. 4, Table 2). Plant cells (H1 and K3 genotypes) were also challenged with organic extracts from *A. dauci* growing in various conditions. The results were similar to those obtained previously after treatment with rA or oA: cells from the susceptible H1 cultivar did not undergo embryogenesis, while the resistant K3 cells did (Table S1). These extracts included a 5 µM zinniol-containing organic extract obtained from a fungal culture grown 12 days in anoxic conditions.

### Plant cell reaction to Alternaria dauci exudates and zinniol: cytoplasmic esterase activity

Cell suspensions underwent the same set of treatments as in the cell somatic embryogenic ability experiment. Esterase activity was measured 48 h after treatment of cell suspensions. In a first step, the activity was modeled using ANOVA followed by least significant difference (LSD) multiple comparison (Table 3). Two different ANOVAs were performed, one based on the whole dataset while taking cultivar × treatment combinations as a factor (h1), and another whereby the activity was modeled separately in each cultivar while taking treatments as a factor (h2). Classically, ANOVA on whole dataset are preferred, but overall variance was influenced by the cultivar, thus breaching homoscedasticity assumptions. Both methods yielded very similar results, as presented in Table 3. Where not explicitly indicated, only homogeneous groups obtained using separate ANOVAs are discussed here.

**Table 3:** Influence of cultivar, fungal exudates fractions and zinniol on cell suspension esterase activity.

Bolero			H1			H4			I2			K3			
Treatment	ESA^1^	h1^2^	h2^3^	ESA	h1	h2	ESA	h1	h2	ESA	h1	h2	ESA	h1	h2
rA^4^	602	b	b	229	nopqrs	cde	211	opqrs	cd	415	cdefghi	b	315	ijklmno	cde
rM	388	cdefghijk	c	288	klmnopq	bc	190	qrs	cd	343	fghijklm	cd	284	klmnopq	ef
aA	484	c	c	256	mnopqrs	bcde	168	rs	cd	486	c	a	402	cdefghij	a
aM	422	cdefghi	c	300	jklmnop	bc	149	s	d	346	efghijklm	cd	373	defghijkl	ab
oA	770	a	a	185	qrs	e	455	cde	b	282	klmnopq	de	221	opqrs	g
oM	430	cdefgh	c	193	pqrs	de	446	cdefg	b	224	opqrs	e	218	opqrs	g
C	436	cdefgh	c	314	ijklmno	b	339	ghijklm	bc	283	klmnopq	de	285	klmnopq	def
DMSO	482	c	c	279	lmnopq	bc	481	c	b	283	klmnopq	de	256	mnopqrs	fg
z1	771	a	a	449	cdef	a	720	a	a	440	cdefgh	ab	340	fghijklm	bcd
z2	602	b	b	262	mnopqr	bcde	676	ab	a	350	efghijklm	c	351	efghijklm	abc
z3	463	cd	c	265	lmnopqr	bcd	490	c	b	337	hijklmn	cd	359	defghijklm	abc

Carrot cell suspensions with six different genotypes were tested for esterase specific activity in the presence of fungal extracts and toxins.

1 ESA is for Esterase Specific Activity, expressed in nmol min^−1^ mg(prot)^−1^.

2 Activities with the same letter are not significantly different. h1 homogeneity groups were obtained by a single ANOVA analysis of all the results followed by LSD multiple comparisons.

3 Activities with the same letter are not significantly different. h2 homogeneity groups were obtained by a separate ANOVA analysis of the results for each cultivar followed by LSD multiple comparisons.

4 The treatments were as follows: rA: *Alternaria dauci* (strain FRA017) fungal culture raw extract; rM: uninoculated medium raw extract; aA: *A. dauci* fungal culture aqueous extract; aM: uninoculated medium aqueous extract; oA: *A. dauci* fungal culture organic extract; oM: uninoculated medium organic extract; DMSO: DMSO solution at a concentration corresponding to oM, z1, z2 and z3 treatments; z1: 0.025 µM zinniol; z2: 10 µM zinniol; z3: 500 µM zinniol. C: no treatment.

Cell suspensions treated with uninoculated fungal medium raw extract (rM) did not show significant variations in esterase activity as compared to untreated cells. In contrast, the raw fungal extracts (rA) had significant effects. When compared with the untreated control, esterase activity was significantly lower in the susceptible cultivar H1, and significantly higher in the resistant cultivars Bolero and I2. Non-significant variations were observed in K3 (resistant, rise) and H4 (intermediate, drop). Similar trends were observed when comparing rA and rM, except that the decreased activity in H1 was not significant. Cell suspensions treated with uninoculated fungal medium organic extract (oM) did show any significant variation in esterase activity as compared to untreated cells. The oA effects were thus compared with oM. In these conditions, a significant increase was observed in the resistant cultivar Bolero. Non significant variations were noted in the other cultivars: an increase for I2, and minute variations for genotypes K3, H1 and H4. Cell suspensions treated with aM showed significantly different activities than control in H4 (drop) and K3 (rise). Compared to aM, the aA effects were as follows: a significant increase in the resistant genotype I2, non-significant increases in genotypes K3, H4 and Bolero, and non-significant decrease in the susceptible genotype H1. DMSO treated cell suspensions did not show significant esterase activity variations, with the exception of H4, where a sharp increase was observed. This variation was not significant in the H4 separate ANOVA, but was significant when ANOVA was performed on the whole dataset. The z1 treatment led to a highly significant increase in esterase activity, irrespective of the cultivar considered. The z2 treatment led to a significant increase in specific esterase activity, except for H1, where no significant variation was observed. The z3 treatment led to non-significant variations in esterase activity, except for K3 (significant increase).

**Figure 5:**
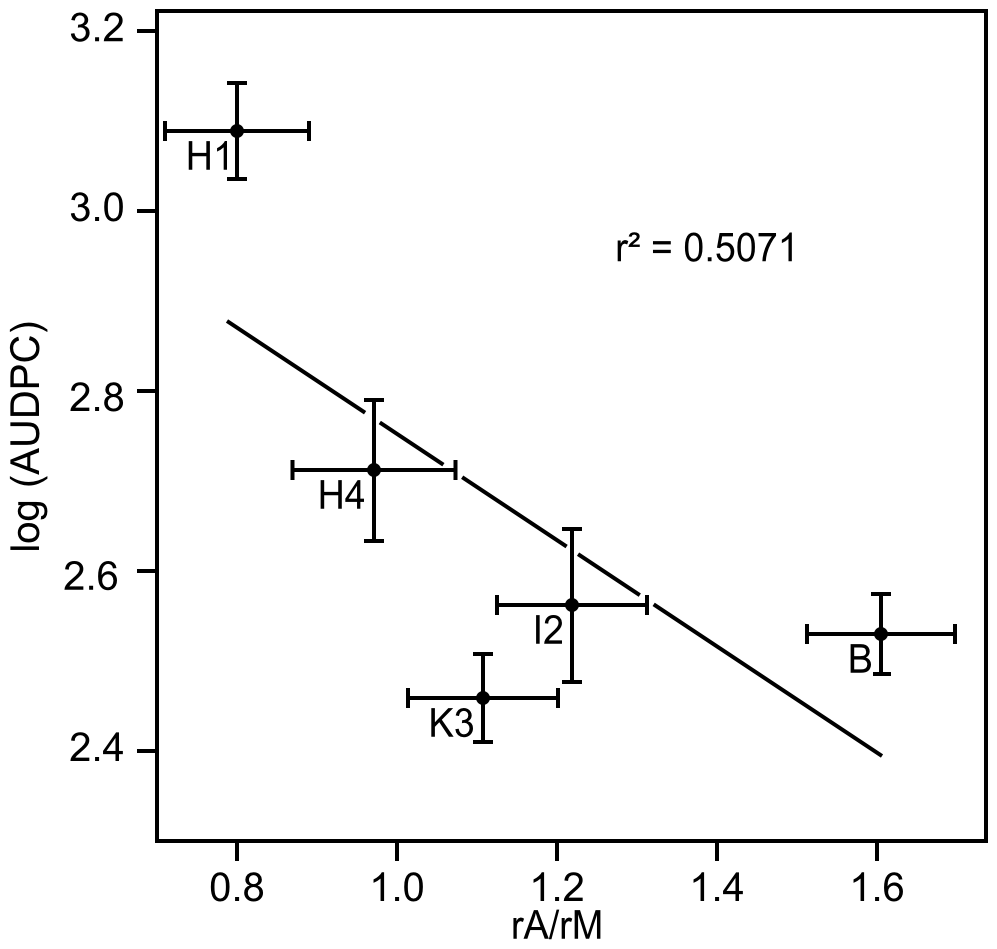
Correlation between cell suspension reactions to *Alternaria dauci* raw extracts and whole plant resistance to the *A. dauci* fungus. log(AUDPC) data, calculated from visual assessments, are the same than in Table 1. The same genotypes were also tested for esterase activity in the presence of fungal (rA) or uninoculated medium (rM) raw extracts. rA/rM denotes esterase activity variations due to the presence of fungal extracts. A negative correlation coefficient (r = −0.7221, r^2^ = 0.5071) was noted between rA/rM and log(AUDPC).

**Figure 6:**
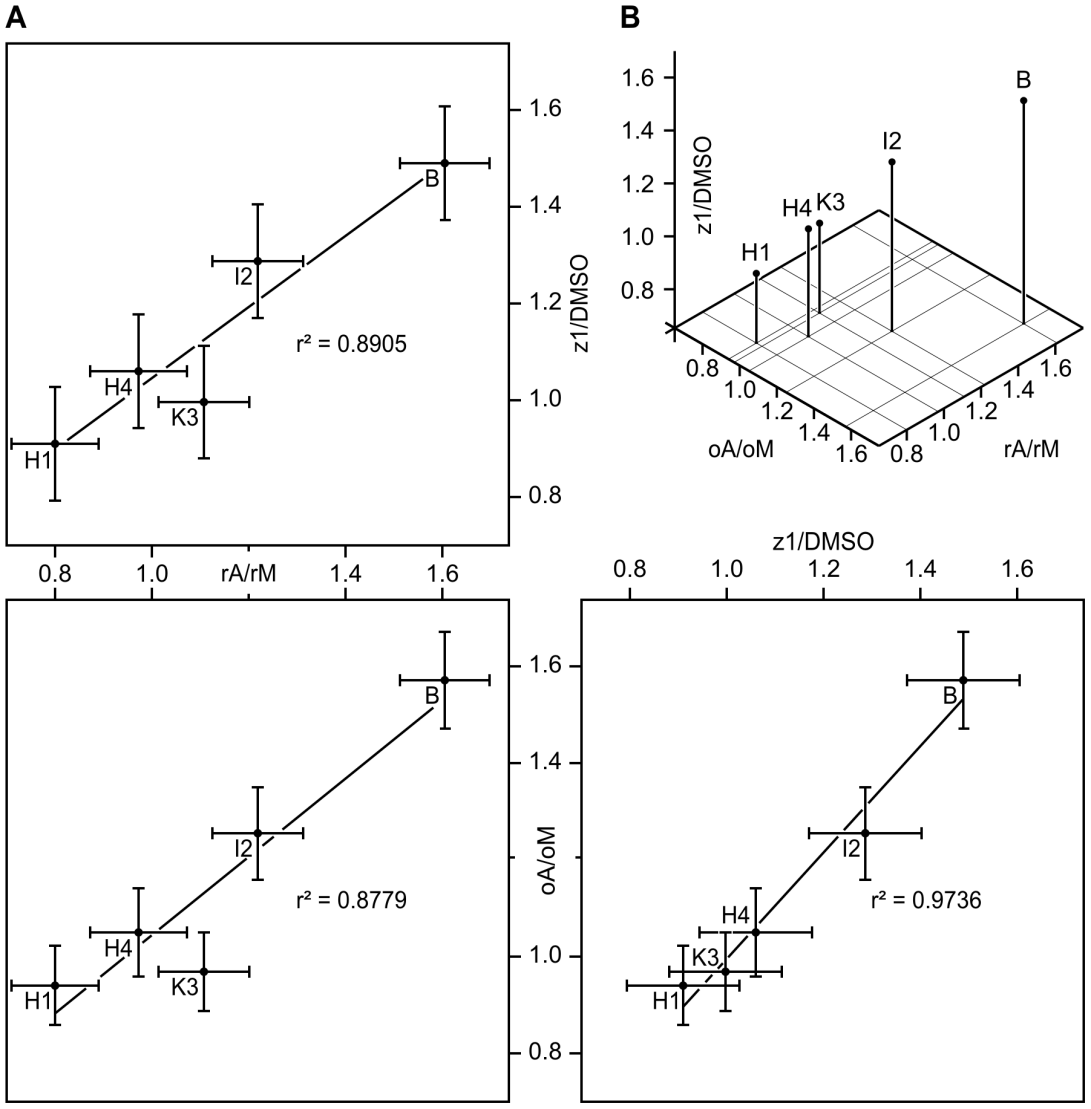
Correlations between cell suspension reactions to *Alternaria dauci* raw extracts, organic extracts and low zinniol concentrations. Five carrot genotypes were tested for their metabolic activity when *A. dauci* raw (rA) or organic (oA) extract was added to the plant culture medium. The same experiments were conducted while adding uninoculated medium raw (rM) or organic (oM) extract and 0.025 µM zinniol to DMSO (z1) or DMSO. rA/rM denotes plant cell esterase activity variations due to the presence of fungal raw extracts, oA/oM denotes plant cell esterase activity variations due to the presence of fungal organic extracts, and z1/DMSO denotes plant cell esterase activity variations due to the presence of 0.025 µM zinniol in the medium. A: correlation plots of rA/rM, oA/oM and z1/DMSO by pairs. Bars represent standard errors. The three paired correlated activity indices presented here correspond to the most significant r^2^ values (see Table 4). B: 3D correlation plot of rA/rM, oA/oM and z1/DMSO.

Specific activity ratios were calculated in order to isolate the *A. dauci* exudate and toxin effects from the fungal growth medium and solvent effects: *A. dauci* exudates versus uninoculated medium (rA/rM, oA/oM and aA/aM), or zinniol versus DMSO (z1/DMSO, z2/DMSO and z3/DMSO). Correlations between these ratios and between rA/rM and AUDPC were investigated. As expected, a negative correlation (r = −0.7121, r^2^ = 0.5071) was obtained between rA/rM and AUDPC. Indeed, a trend was noted when AUDPC was plotted against rA/rM (Fig. 5): the susceptible cultivar H1 showed the highest AUDPC and the lowest rA/rM ratio, while the resistant cultivar Bolero combined the highest rA/rM ratio with a very low AUDPC. H4 and I2 seemed intermediate between these two extremes. One of the resistant genotypes (K3) was out of line with the main trend: although it was quite resistant towards *A. dauci*, it did not show strong esterase relative activity in the presence of rA. When K3 was removed, the r^2^ increased to 0.7038. When the relative enzymatic activities were compared for the different fungal fractions or toxin concentrations tested, only three revealed a statistically significant correlation (Table 4), and the highest correlation was between oA/oM and z1/DMSO (r^2^ = 0.9736, p = 0.184%). Similarly, rA/rM was closely correlated with z1/DMSO (r^2^ = 0.8905, p = 1.59%) and oA/oM (r^2^ = 0.8779, p = 1.88%). When plotted against each other, these ratios showed a close correlation (Fig. 6). Once again, K3 seemed to be slightly out of line with the main trend, with z1/DMSO and oA/oM values lower than expected in comparison to the rA/rM values.

**Table 4:** Correlation coefficients for esterase activity ratios.

	z3/DMSO	z2/DMSO	z1/DMSO	oA/oM	aA/aM
rA/rM	0.0217	0.0026	**0.8906**	**0.8779**	0.4037
aA/aM	0.1610	0.0560	0.5327	0.3723	
oA/oM	0.0463	0.0195	**0.9736**		
z1/DMSO	0.0122	0.0240			
z2/DMSO	0.5734				

Carrot cell suspensions with five different genotypes were tested for esterase relative specific activity in the presence of fungal extracts and toxins. The treatments were as follows: rA: *A. dauci* (strain FRA017) fungal culture raw extract; rM: uninoculated medium raw extract; aA: *A. dauci* fungal culture aqueous extract; aM, uninoculated medium aqueous extract; oA: *A.dauci* fungal culture organic extract; oM: uninoculated medium organic extract; DMSO: DMSO solution at a concentration corresponding to oM, z1, z2 and z3 treatments; z1: 0.025 µM zinniol; z2: 10 µM zinniol; z3: 500 µM zinniol. Correlation coefficients corresponding to significant (α = 0.05) linear regressions are in bold.

These results partially confirmed the data obtained in the embryogenesis experiment: a negative correlation was found between infected plant disease extent and relative esterase activity in the presence of fungal raw extracts. Reactions to fungal raw and organic extracts were almost the same. Nevertheless, there were several marked differences between these two datasets. First, there was a strong correlation between the low zinniol concentration effect and the raw or organic extract effect. Second, although raw and organic extracts did effectively block embryogenesis amongst susceptible cultivars, esterase activity was not markedly affected by these extracts in the susceptible cultivar H1 after 48 h of exposure.

**Figure 7:**
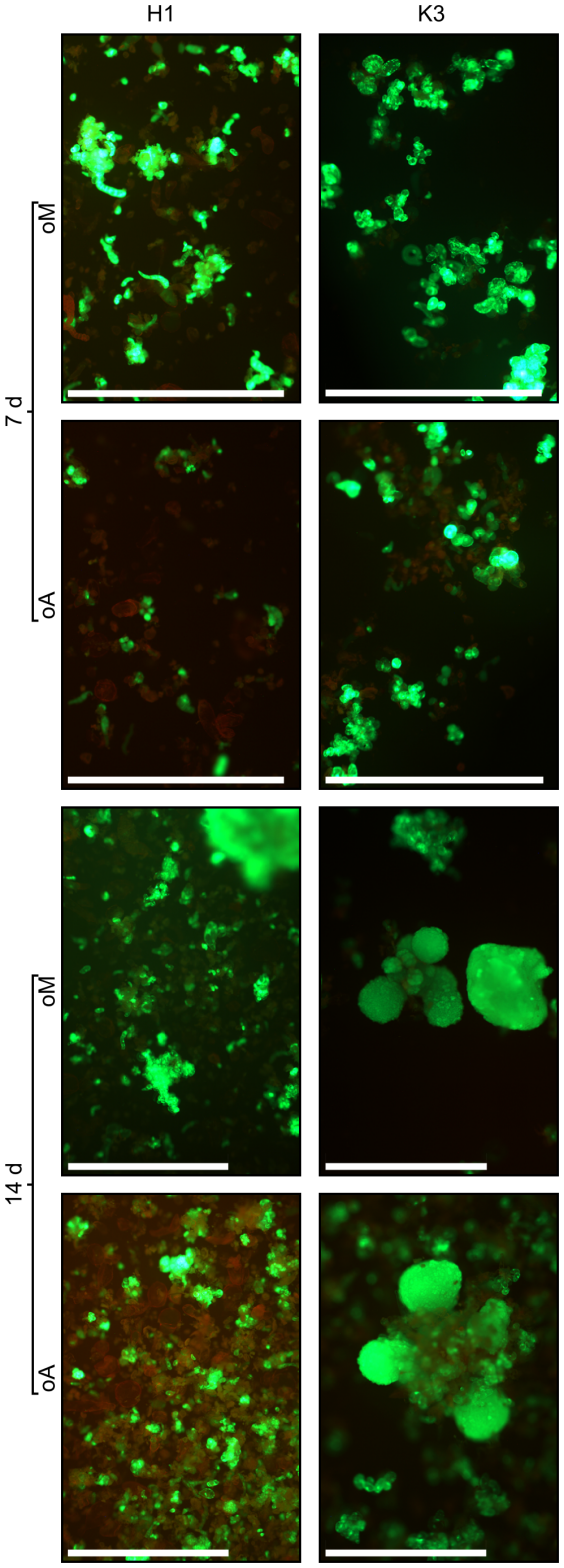
Toxicity and resistance evaluations using fluorescence microscopy. Liquid cell cultures from two carrot genotypes were tested for mortality and metabolic activity when *Alternaria dauci* organic extract (oA) was added to the plant culture medium. The same experiments were conducted while adding uninoculated medium organic extract (oM). Seven and 14 days after adding extracts, membrane integrity and cell viability were evaluated by microscopy using a double staining method with fluoresceine diacetate (FDA) and propidium iodide (IP). The images shown are representative of results obtained from three independent experiments. oA treated K3 cell esterase activity, survival and embryogenesis could not be differentiated from oM treated cells. At 7 days, mortality was somewhat higher and esterase activity lower in oA- than oM-treated H1 cells. At 14 days, much greater observed differences followed a similar trend. High mortality was visible in oA treated H1 cells compared to oM-treated cells. Moreover, proembryogenic masses were visible in oM-treated H1 cultures, and not in oA treated cultures.

In order to investigate this apparent discrepancy, we used microscopy to assess H1 and K3 cell survival and esterase activity rates after 7 and 14 days of exposure to either fungus (oA) or the uninoculated medium (oM) organic phase (Fig. 7). oA treated K3 cell esterase activity, survival and embryogenesis could not be differentiated from oM treated cells. At 7 days, mortality was somewhat higher and esterase activity lower in oA- as compared to oM-treated H1 cells. The much greater differences observed at 14 days followed a similar trend. High mortality was noted amongst oA treated H1 cells as compared to oM-treated cells. Moreover, proembryogenic masses were visible in oM-treated H1 cultures, and not in oA treated cultures.

## Discussion

The aim of this study was to investigate the role of fungal toxins in both pathogenicity and resistance in the carrot-*A. dauci* interaction. Since *A. dauci* toxins are not fully known [29], we opted to confront *in vitro* cultured carrot cells with raw fungal extracts. A differential response to phytotoxicity was clearly demonstrated between susceptible and partially resistant carrot genotypes after fungal exudate treatment of plant embryogenic cultures. A close correlation was noted between the resistance to *A. dauci* at the whole plant level and resistance to fungal exudates at the cellular level.

The toxicity of raw and organic fungal extracts was clearly noted, while no toxic reaction of embryogenic cultures was obtained after treatment with the aqueous extract. If toxic metabolites were present in this aqueous extract, their concentrations were likely too low to induce toxic effects. Peptidic HSTs have previously been described in other pathosystems involving fungi: *Stagonospora nodorum*, *Pyrenophora tritici-repentis* and two *Alternaria* species (AB-toxin in *A. brassicicola*, AP-toxin in *A. panax*, for a review, see [47]). The production of such toxic peptides in *A. dauci* exudates has, to our knowledge, never been reported. In our study, zinniol, a putative NHST used at physiological concentrations (10 µM), did not exhibit toxicity towards carrot embryogenic cultures. Moreover, zinniol was not detected in exudates collected from 48 h fungal cultures. Five µM zinniol was produced by the same fungal strain in exudates from a 12 day culture under anoxic conditions, which is in line with the findings of Barash et al. [24]. This highly suggests that: (i) zinniol was not responsible for the phytotoxic reactions observed after treatment with the organic extract, and (ii) one or several unknown toxic hydrophobic metabolites were produced by *A. dauci*.

In previous studies, zinniol toxicity was evaluated by direct application of this compound on leaves of different plant species (including carrot) at relatively high concentrations ranging from 150 µM to 1 mM [24], [26], [48]. Application of 500 µM zinniol to *Tagetes erecta* cell suspensions was deleterious for the cultures [28]. Using the same zinniol concentration, we observed a similar response from carrot cell suspensions irrespective of the plant genetic background. However, due to the high zinniol concentrations used in the papers cited above, the results obtained at whole plant or cellular levels probably overestimated the role of zinniol as a phytotoxin. This bias seems to be absent in papers investigating the activity of other phytotoxins. For example, in the *Stemphylium solani*- *Allium sativum* pathosystem [22], necrotic lesions were observed on leaves of a susceptible garlic genotype using a purified toxin (SS-toxin) at 11 µg mL^−1^ concentration from a 21 day fungal culture filtrate. In the present study, no phytotoxic reactions were observed using 10 µM zinniol (3 µg mL^−1^). Consequently, zinniol is probably not a phytotoxin as previously suggested by Qui et al. [28]. By comparison, we obtained toxic effects on carrot embryogenic cultures treated with the organic extract at 7.5 µg mL^−1^ concentration (25% of the original fungal culture medium). Moreover, the HPLC spectra indicated that no dominant hydrophobic metabolite was present. These two combined results suggest the production by *A. dauci* of hydrophobic compounds at least 5-fold more toxic than zinniol to carrot cells.

In this study, carrot *in vitro* cell suspensions from several genotypes were challenged with fungal extracts and zinniol. Compound toxicity and genotype resistance were evaluated on the basis of cell viability and embryogenic ability. In several other studies, plant cell reactions to compounds produced by pathogenic fungi were investigated using *in vitro* cell suspension cultures. Cultured grapevine cell defense-related compound production was enhanced by adding autoclaved *Phaeomoniella clamydospora* biomass [49]. Similarly, when challenged with two distinct *Botrytis cinerea* elicitors (botrycin and cinerein), cultured grapevine cells showed defense reactions that differed depending on the tested elicitor [50]. Conversely, fungal toxins from *Rhizoctonia solani* and *Sarocladium oryzae* were shown to inhibit defense-related compounds in rice cell suspensions [51]. A link between plant pathogen partial resistance and toxin resistance has been suggested in the *Allium sativum* –*Stemphylium solani* pathosystem [22] but, to our knowledge, the present study is the first example where *in vitro* cell viability and embryogenic ability were used as an indicator of fungal toxin plant resistance. In our study, we adapted cell viability measurement methods based on measuring esterase activity using FDA as a substrate to carrot cell suspension cultures. Cell viability is classically measured using counting methods in which viable and nonviable cell numbers are compared. Nevertheless these methods lack accuracy because of the weight and clumpiness of cultured plant cells. Since FDA fluorescence was proposed by Rotman and Papermaster [52] as a way of measuring esterase activity, this procedure has been very widely used to measure cell viability and activity, e.g. in *Medicago truncatula* [37] or soil microorganisms [44]. We also adapted the microscopy techniques proposed by Vitecek *et al.* [43] using both FDA green fluorescence and propidium iodide red fluorescence in damaged cells as a good indicator of viability.

Although this study was not aimed at assessing the kinetics of the effects of fungal toxins on carrot cells, observations were performed at different times: esterase activity quantification was performed 48 h after adding extracts. Microscopic observations were performed after 7 days and 14 days of exposure, while embryogenesis was observed after 21 days of exposure. Overall, these results suggest a long-term effect of the fungal extract: at 48 h, the average esterase activity of susceptible H1 cells relative to that of unexposed cells was 80% (Fig. 5). Differences with respect to the negative control were noted after 7 days, and they were more clearcut after 14 days (Fig. 7). Nevertheless, some cells were still alive. At 21 days, no embryogenesis was visible, and only debris was observed (Fig. 4A). Since no further variations were noted after several more weeks, we assumed that no more living cells were present. This should perhaps be considered in the light of the fact that, even under very favorable conditions (24°C, 100% RH, in susceptible cultivars such as Presto), the first symptoms were only visible 7 days after inoculation. In favorable conditions, other plant fungal pathogens cause visible symptoms earlier (often within 72 h, e.g. with *Magnaporthe grisea*, Fig. 3 in [40], *Botrytis cinerea* (see Fig. 1 in [53]), or *Alternaria brassicicola* (see Fig. 8 in [54]).

Amongst resistant cultivars I2 and Bolero, esterase activity was enhanced after 48 h of exposure to fungal raw extract, organic extract, and low zinniol concentrations. These results surprised us as we expected to detect toxicity through a drop in esterase activity, and resistance through the absence of such a drop in resistant cultivars, as was observed in resistant cultivar K3. As an afterthought, a rise in esterase activity could perhaps be interpreted as a plant resistance reaction. FDA enters plant cells where it can be hydrolyzed by various enzymes, including proteases, lipases and esterases [52]. Such hydrolytic enzymes can be linked with plant defense mechanisms through mobilization of the primary energy metabolism, reducing ability and carbon skeleton for defense [55]. Under that hypothesis, the higher metabolic activity of Bolero and I2 cells could be explained by the fact that, in these cultivars, plant cells are able to detect hydrophobic compounds produced by fungi that include zinniol. The data presented here are not out of line with this interpretation. A high correlation was found between esterase activities in the presence of organic fungal exudates and the low zinniol concentration (Fig. 6). As these effects were measured 48 h after plant cell exposure to zinniol or organic extract, the low concentrations of zinniol produced by *A. dauci* might be involved in the plant response right after the onset of the plant-pathogen interaction. Zinniol was not found in our fungal exudates, but its presence at very low concentrations could not be ruled out. Besides, zinniol was detected in infected plant tissues in at least two different pathosystems at early stages of plant infection: 2 days after sunflower infection by *Phoma macdonaldii* [27] and 12 h after carrot infection by *A. dauci* [56]. More generally, elicitors are often described as small secreted proteins or polymers, but there seem to be other cases where fungal secondary metabolites [57], or more generally small molecules [58] play such a role.

In conclusion, three main insights emerged from the presented data: (i) strongly phytotoxic compounds are present in the organic phase of *A. dauci* exudates, (ii) zinniol is not the main phytotoxic compound produced by *A. dauci*, and (iii) carrot resistance to *A. dauci* involves cellular resistance to these compounds. Our study also raised new questions, especially concerning the nature of the hydrophobic toxic compounds present in the organic phase. *Alternaria dauci* aggressiveness varies strongly depending on the strain [33]. It would be interesting to determine if these variations are linked with quantitative or qualitative variations in the production of those compounds in fungal strains. Moreover, the role of zinniol in the carrot-*A. dauci* interaction should be redefined.

## Supporting Information

Figure S1 **HPLC analysis of zinniol stability in different solutions.** HPLC chromatograms were obtained from different 10 µg zinniol samples after an incubation of one week at room temperature. **A**: Zinniol incubated in a deuterated DMSO-aqueous buffer at pH 5.6. **B**: Zinniol incubated in B5 Gamborg medium (0.4% DMSO, pH 5.8). **C**: Zinniol incubated in CDCl_3_. In A and B, one strong peak is visible at 7.015 minutes, corresponding to zinniol expected retention time. In C, a small peak is visible at the same retention time. Other peaks are visible at 9.63, 14.33, 14.55, 15.30 and 16.40 minutes retention time. AU: Absorption Units (optical density) at 233 nm.
(TIF)

Table S1 **Influence of culture medium and anoxia on fungal exudates organic extracts toxicity.** Carrot cell suspensions with two different genotypes were tested for embryogenesis in the presence of fungal extracts. Embryogenesis was assessed 4 weeks after treatment. ^1^Treatments were as follows: C: no treatment, DMSO: DMSO solution at the same concentration than in organic extracts. Organic extracts from *Alternaria dauci* (strain FRA017) fungal culture grown in the following conditions: oA: 48 h shaking in carrot juice medium, oA4d: 96 h shaking in carrot juice medium, oAV: 72 h shaking in V8 medium, oAVa: 12 days no shaking (anoxia) in V8 medium, oC uninoculated carrot medium. ^2^The signs are as follows: (−) no embryogenesis was visible and cells were damaged, (+) early-stage embryogenic masses were visible, (++) embryos were present, (+++) embryogenesis was profuse.
(DOCX)
