# Supplementary figures and images for: Partial Resistance of Carrot to Alternaria dauci Correlates with In Vitro Cultured Carrot Cell Resistance to Fungal Exudates
Source: PLoS One. 2014 Jul 1;9(7):e101008. doi: 10.1371/journal.pone.0101008 (PMC4077726; doi:10.1371/journal.pone.0101008)

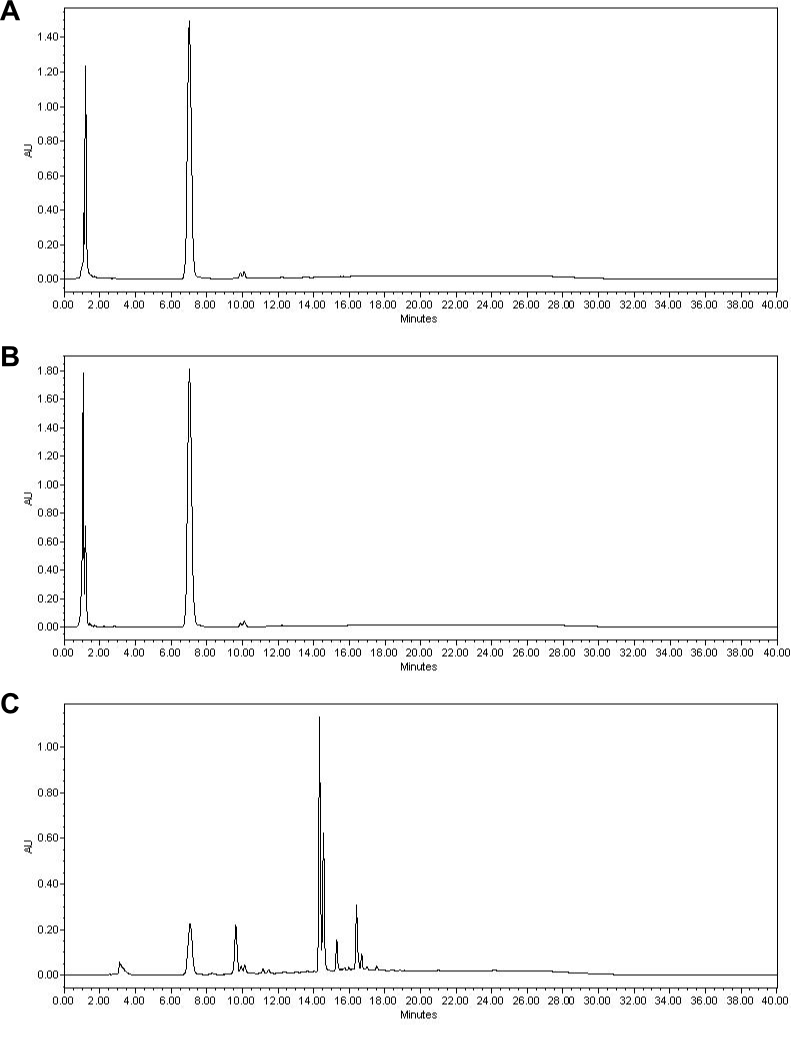

Supplement: Figure S1 — HPLC analysis of zinniol stability in different solutions. HPLC chromatograms were obtained from different 10 µg zinniol samples after an incubation of one week at room temperature. A: Zinniol incubated in a deuterated DMSO-aqueous buffer at pH 5.6. B: Zinniol incubated in B5 Gamborg medium (0.4% DMSO, pH 5.8). C: Zinniol incubated in CDCl3. In A and B, one strong peak is visible at 7.015 minutes, corresponding to zinniol expected retention time. In C, a small peak is visible at the same retention time. Other peaks are visible at 9.63, 14.33, 14.55, 15.30 and 16.40 minutes retention time. AU: Absorption Units (optical density) at 233 nm. (TIF) [file pone.0101008.s001.tif]
