# Supplementary material for: Partial Resistance of Carrot to Alternaria dauci Correlates with In Vitro Cultured Carrot Cell Resistance to Fungal Exudates
Source: PLoS One. 2014 Jul 1;9(7):e101008. doi: 10.1371/journal.pone.0101008 (PMC4077726; doi:10.1371/journal.pone.0101008)
Supplement: Table S1 — Influence of culture medium and anoxia on fungal exudates organic extracts toxicity. Carrot cell suspensions with two different genotypes were tested for embryogenesis in the presence of fungal extracts. Embryogenesis was assessed 4 weeks after treatment. 1Treatments were as follows: C: no treatment, DMSO: DMSO solution at the same concentration than in organic extracts. Organic extracts from Alternaria dauci (strain FRA017) fungal culture grown in the following conditions: oA: 48 h shaking in carrot juice medium, oA4d: 96 h shaking in carrot juice medium, oAV: 72 h shaking in V8 medium, oAVa: 12 days no shaking (anoxia) in V8 medium, oC uninoculated carrot medium. 2The signs are as follows: (−) no embryogenesis was visible and cells were damaged, (+) early-stage embryogenic masses were visible, (++) embryos were present, (+++) embryogenesis was profuse. (DOCX) [file pone.0101008.s002.docx]

| Treatment | Carrot genotype | |
| --- | --- | --- |
|  | H1 | K3 |
| oA^1^ | –^2^ | +++ |
| oA4d | – | +++ |
| oAV | – | +++ |
| oAVa | – | +++ |
| C | ++ | +++ |
| DMSO | + | ++ |
| oC | + | +++ |
